# A multicenter study assessing the prevalence of germline genetic alterations in Chinese gastric-cancer patients

**DOI:** 10.1093/gastro/goab020

**Published:** 2021-05-29

**Authors:** Yin-Jie Zhang, Yang Yang, Qing Wei, Ting Xu, Xiao-Tian Zhang, Jing Gao, Si-Yi Tan, Bao-Rui Liu, Jing-Dong Zhang, Xiao-Bing Chen, Zhao-Jie Wang, Meng Qiu, Xin Wang, Lin Shen, Xi-Cheng Wang

**Affiliations:** 1 Department of Gastrointestinal Oncology, Key Laboratory of Carcinogenesis and Translational Research (Ministry of Education/Beijing), Peking University Cancer Hospital & Institute, Beijing, P. R. China; 2 Department of Medical Oncology, Sichuan Cancer Hospital & Institute, Sichuan Cancer Center, School of Medicine, University of Electronic Science and Technology of China, Chengdu, Sichuan, P. R. China; 3 Comprehensive Cancer Center of Drum Tower Hospital, Medical School of Nanjing University & Clinical Cancer Institute of Nanjing University, Nanjing, Jiangsu, P. R. China; 4 Department of Medical Oncology, Zhejiang Cancer Hospital, Hangzhou, Zhejiang, P. R. China; 5 Department of Medical Oncology, Cancer Hospital of China Medical University, Liaoning Cancer Hospital & Institute, Shenyang, Liaoning, P. R. China; 6 Department of Gastroenterology and Medical Oncology, Henan Cancer Hospital (Affiliated Cancer Hospital of Zhengzhou University), Zhengzhou, Henan, P. R. China; 7 Department of Oncology, Henan Provincial People’s Hospital, Zhengzhou, Henan, P. R. China; 8 Department of Medical Oncology, Cancer Center, the State Key Laboratory of Biotherapy, West China Hospital, West China Medical School, Sichuan University, Chengdu, Sichuan, P. R. China; 9 State Key Laboratory of Cancer Biology, Xijing Hospital of Digestive Disease, Fourth Military Medical University, Xi’an, Shaanxi, P. R. China

**Keywords:** familial gastric cancer, next-generation sequencing, germline mutation, cancer-predisposition gene

## Abstract

**Background:**

Approximately 10% of patients with gastric cancer (GC) have a genetic predisposition toward the disease. However, there is scant knowledge regarding germline mutations in predisposing genes in the Chinese GC population. This study aimed to determine the spectrum and distribution of predisposing gene mutations among Chinese GC patients known to have hereditary high-risk factors for cancer.

**Methods:**

A total of 40 GC patients from 40 families were recruited from seven medical institutions in China. Next-generation sequencing was performed on 171 genes associated with cancer predisposition. For probands carrying pathogenic/likely pathogenic germline variants, Sanger sequencing was applied to validate the variants in the probands as well as their relatives.

**Results:**

According to sequencing results, 25.0% (10/40) of the patients carried a combined total of 10 pathogenic or likely pathogenic germline variants involving nine different genes: *CDH1* (*n *=* *1), *MLH1* (*n *=* *1), *MSH2* (*n *=* *1), *CHEK2* (*n *=* *1), *BLM* (*n *=* *1), *EXT2* (*n *=* *1), *PALB2* (*n *=* *1), *ERCC2* (*n *=* *1), and *SPINK1* (*n *=* *2). In addition, 129 variants of uncertain significance were identified in 27 patients.

**Conclusions:**

This study indicates that approximately one in every four Chinese GC patients with hereditary high risk factors may harbor pathogenic/likely pathogenic germline alterations in cancer-susceptibility genes. The results further indicate a unique genetic background for GC among Chinese patients.

## Introduction

Gastric cancer (GC) is the third most common cause of cancer-related mortality worldwide [1]. Approximately 10% of GC cases are associated with strong familial clustering and can be attributed to genetic predisposition [2, 3]. Moreover, it has been established that 1.0%–3.0% of GC cases occur due to inherited cancer-predisposition syndromes, including hereditary diffuse gastric cancer (HDGC), Lynch syndrome (LS) [4–6], Li–Fraumeni syndrome (LFS) [7, 8], Peutz–Jeghers syndrome (PJS) [9–11], hereditary breast and ovarian cancer (HBOC) [12, 13], *MUTYH*-associated adenomatous polyposis (MAP) [14], familial adenomatous polyposis (FAP) [15–17], juvenile polyposis syndrome (JPS) [18, 19], and PTEN hamartoma tumor syndrome [20]. Since the discovery of the GC susceptibility gene *CDH1* in 1998 [21], >20 GC-associated susceptibility genes have been identified, including *CDH1*, *MLH1*, *MSH2*, *MSH6*, *PMS2*, *EPCAM*, *TP53*, *STK11*, *BRCA1*, *BRCA2*, *MUTYH*, *APC*, *SMAD4*, *BMPR1A*, and *PTEN* [22]. Nevertheless, because of the low incidence of individual predisposing gene mutations, conventional approaches such as Sanger sequencing may yield false-negative results owing to the limited sequencing coverage. However, with the widespread application of next-generation sequencing (NGS), multiple-gene panel testing is now commercially and clinically available for cancer-risk assessment. In particular, multiple-gene sequencing of germline DNA can be used to identify novel variants and risk alleles of varying penetrance for *CDH1*-negative families that meet HDGC criteria.

In general, the marked differences in ethnicity, diet, and living habits between Chinese and Caucasian populations suggest that Western genetic-screening guidelines may not be suitable for Eastern populations. To date, research on hereditary GC in the Chinese population is scarce. Nonetheless, it is important to identify patients with genetic aberrations because such mutations may influence clinical management, yet the underlying genetic factors conferring susceptibility to GC remain largely unknown. Accordingly, this study aimed to explore the frequency and spectrum of predisposing germline gene variants among Chinese GC patients with hereditary high risk factors for cancer. For probands harboring pathogenic or likely pathogenic germline variants, Sanger sequencing was applied to validate the variants in both the probands and their family members.

## Patients and methods

### Study population

Between January 2017 and August 2018, gastric-adenocarcinoma patients with hereditary high risk factors were recruited from seven hospitals throughout six provinces in China (Supplementary Table 1). Patients who met one of the following criteria for high risk were included: (i) onset age ≤30 years, regardless of family history; (ii) onset age ≤35 years and GC histologically classified as signet ring cell carcinoma (SRCC) or mucinous adenocarcinoma, regardless of family history; (iii) onset age ≤50 years and at least one first-degree relative diagnosed with malignant tumors; (iv) at least two first- or second-degree relatives diagnosed with malignant tumors, with at least one first-degree relative included; (v) diagnosed with more than two primary malignant tumors, with one having an onset age ≤50 years; and (vi) tissue specimens showing microsatellite instability or deficient mismatch repair (MMR).

This study was approved by the Ethics Committee of Peking University Cancer Hospital and the relevant ethics committees of each of the participating centers. All procedures were performed in accordance with the ethical standards of the respective committees on human experimentation (institutional and national) and with the Helsinki Declaration of 1964 and its later versions. All patients and their family members provided written informed consent to participate.

### Sequencing panel design

The solution-phase panel was designed to cover all exons (including parts of the introns) of 171 cancer-predisposing genes selected following a thorough literature review and a review of unpublished data (Supplementary Table 2).

### NGS, bioinformatics, and variant filtering

The processes of genomic DNA extraction, NGS, bioinformatic analysis, and variant filtering and annotation were supported by BGI Genomics (Shenzhen, China) and performed as a previous study has described [23].

### Statistical analyses

Statistical analyses were performed with SPSS Statistics 21.0 software (IBM Corp., Armonk, NY, USA). For continuous variables (age at diagnosis), data with non-normal distributions are presented as medians with interquartile ranges; rank tests were used for all other analyses. Categorical variables are presented as ratios. Differences in mutation rates between groups were compared and analysed with the chi-square (χ^2^) test. *P *<* *0.05 was considered statistically significant.

## Results

### Patient cohort and characteristics

Forty GC patients and their families were recruited from seven medical centers across six provinces in China (Supplementary Table 2). The clinical characteristics of the patients are presented in Table 1. Associations between these six conditions and pathogenic/likely pathogenic variants were analysed, giving a clue for optimizing the selection of Chinese GC patients with hereditary high risk factors (Supplementary Table 3). The median age at initial GC diagnosis was 37.5 years (range, 24–76 years). Among the 40 GC patients, 26 (65.0%) showed an advanced disease stage (III to IV); 22 (55.0%) were diagnosed with adenocarcinoma, 16 (40.0%) with SRCC, and 2 (5.0%) with mucinous adenocarcinoma. Overall, 29 (72.5%) patients had a family history of malignancies. The detailed baseline information of the 40 patients is listed in Supplementary Table 4.

**Table 1.** Clinical characteristics of the 40 patients with gastric cancer included in this study

Characteristic	No. of patients (%)
Sex	
Male	23 (57.5)
Female	17 (42.5)
Age at diagnosis (years)	
≤30	16 (40.0)
31–40	6 (15.0)
41–50	2 (5.0)
51–60	12 (30.0)
>60	4 (10.0)
Tumor stage	
I	6 (15.0)
II	8 (20.0)
III	15 (37.5)
IV	11 (27.5)
Histological types	
Adenocarcinoma	22 (55.0)
Mucinous adenocarcinoma	2 (5.0)
Signet ring cell carcinoma	16 (40.0)
Family history	
Yes	29 (72.5)
No	11 (27.5)

### Pathogenic or likely pathogenic germline variants

**Figure 1.**
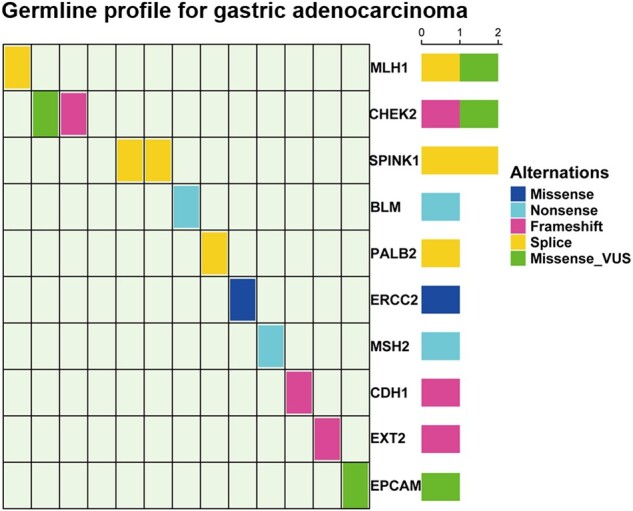
Germline mutations in cancer-susceptibility genes. (A) Distribution of germline mutations in Chinese GC patients. (B) Germline profiles for GC patients with pathogenic/likely pathogenic variants.

Among the 40 patients, 10 (25.0%) were found to carry pathogenic or likely pathogenic variants. Two probands carried MMR pathogenic variants (*MLH1 *=* *1, *MSH2 *=* *1) associated with LS. Pathogenic or likely pathogenic variants of homologous recombination repair genes (*BLM *=* *1, *PALB2 *=* *1, *CHEK2 *=* *1) were detected in three probands. One proband harbored a *CDH1* variant associated with HDGC that was, therefore, likely pathogenic. Another two probands carried likely pathogenic variants of other genes known to be associated with a genetic predisposition toward cancer (*EXT2 *=* *1, *ERCC2 *=* *1). Two patients were identified with *SPINK1* mutations, which have not been reported previously (Figure 1). All patients with pathogenic or likely pathogenic variants are listed in Table 2.

**Table 2.** Details of pathogenic or likely pathogenic variants detected via next-generation sequencing

Family code	Cancer	Sex/age at diagnosis GC (years)	Family history of malignancies	Gene	Transcript ID	DNA	Protein	Variant type	Variant classification	
NG5	GC	M/25	Yes	BLM	NM_000057	c.1105C>T	p.Q369*	Nonsense	Likely pathogenic	
XJ1	GC	M/30	no	PALB2	NM_024675	c.1684 + 1G>A	NA	Splice	Likely pathogenic	
LZ2	MPCC, EC, pleural fibrosarcoma, esophageal leiomyosarcoma	F/45	Yes	MLH1	NM_000249	c.790 + 1G>A	NA	Splice	Pathogenic	
BZ1	GC, BC	F/54	Yes	ERCC2	NM_000400	c.1532G>A	p.R511Q	Missense	Likely pathogenic	
BZ2	GC, renal cancer	M/63	Yes	SPINK1	NM_000267	c.194 + 2T>C	NA	Splice	Pathogenic	
BZ3	GC, EC	F/68	Yes	MSH2	NM_000251	c.610G>T	p.G204*	Nonsense	Pathogenic	
BZ5	GC	M/34	Yes	CDH1	NM_004360	c.1475_1479del GAGTG	p.V493Sfs*42	Frameshift	Likely pathogenic	
BZ10	GC, thyroid cancer	F/37	No	SPINK1	NM_000267	c.194 + 2T>C	NA	Splice	Pathogenic	
BZ13	GC	F/24	Yes	EXT2	NM_000401	c.630delC	p.S211 Lfs*92	Frameshift	Likely pathogenic	
BZ16	GC	M/26	no	CHEK2	NM_007194	c.1553_1554insG	p.S518Rfs*7	Frameshift		Likely pathogenic

GC, gastric cancer; MPCC, multiple primary colorectal carcinoma; CRC, colorectal cancer; EC, endometrial cancer; BC, breast cancer.

The proband of the LZ2 family, who harbored an *MLH1* pathogenic mutation, was diagnosed with seven metachronous tumors. A large proportion of her family members also had colorectal cancer, which is a typical presentation of LS. As verified by Sanger sequencing, her son and two nephews did not carry this variant. The proband of the BZ3 family, carrying a pathogenic mutation in *MSH2*, was diagnosed with GC and endometrial cancer at the age of 68 years. All of her siblings were diagnosed with either colorectal cancer, GC, prostate cancer, or endometrial cancer. Three of her children were verified by Sanger sequencing as being positive for the *MSH2* mutation, and the oldest daughter was diagnosed with breast cancer at 34 years old. The proband of the BZ5 family, who carried the *CDH1* mutation, was 34 years old when diagnosed with GC; his elder brother was diagnosed with GC at the same age, and his mother and nephew harbored the same mutation. For patient BZ1 with subsequent onset of breast cancer and GC, the *ERCC2* mutation coexisted with the *MUTYH* mutation. Although her mother was confirmed to have colorectal cancer, her mother and son did not carry these mutations. The NG5 family proband harboring a *BLM* likely pathogenic mutation was diagnosed with GC at the age of 25 years. The patient had a matrilineal family history of cancer, but his father (and not his mother) carried the same mutation; his mother was diagnosed with breast cancer at 45 years old. The patient from the BZ13 family with early-onset GC harbored an *EXT2* likely pathogenic variant inherited from his mother. The patient had a second-degree relative with GC. The patient from the BZ16 family who carried the *CHEK2* likely pathogenic mutation was diagnosed with GC at the age of 25 years and did not have a family history of cancer. His mother and twin brother had the same variant, as verified by Sanger sequencing, whereas his father and aunt did not carry this variant. The XJ1 family proband harbored a *PALB2* mutation and was diagnosed with GC at 30 years, but there was no family history. The Sanger-sequencing results for his parents revealed that the variant was inherited from his father. The probands of the BZ2 family and BZ10 family each harbored a pathogenic mutation in *SPINK1* and were diagnosed with two primary cancers. The former was diagnosed with renal clear cell carcinoma at the age of 62 and subsequently diagnosed with breast cancer at 63. He had a family history of cancer, but his younger brother and younger sister did not carry the same variant, as verified by Sanger sequencing. The patient from the BZ10 family was diagnosed with thyroid cancer and GC at the age of 31 and 37 years, respectively.

### Clinicopathological associations among mutation carriers

No significant differences in age of onset, family history, or clinical stage were found between patients carrying pathogenic or likely pathogenic variants and those with variants of uncertain significance (VUSs). However, patients with pathogenic or likely pathogenic mutations were more likely to have multiple-onset primary malignancies than those without these mutations (50.0% vs 7.69%, Fisher’s exact test, *P *=* *0.011; Table 3).

**Table 3.** Inclusion criteria for pathogenic/likely pathogenic variants

Criteria	Probands with (likely) pathogenic variants	Ratio in pathogenic group	Ratio in non-pathogenic group	*P*-value (Fisher exact)
(i) Onset age ≤30 years, regardless of family history	NG5, XJ1, BZ13, BZ16	40.00%	36.67%	>0.99
(ii) Onset age ≤35 years and GC histologically classified as signet ring cell carcinoma (SRCC) or mucinous adenocarcinoma, regardless of family history	NA	NA	6.67%	>0.99
(iii) onset age ≤50 years and at least one first-degree relative diagnosed with malignant tumors	NG5.LZ2, BZ5	30.00%	16.67%	0.388
(iv) At least two first- or second-degree relatives diagnosed with malignant tumors, with at least one being a first-degree relative	NG5, LZ2, BZ1, BZ2, BZ3	50.00%	56.67%	0.473
(v) Diagnosed with more than two primary malignant tumors, with onset age of one being ≤50 years	LZ2, BZ1, BZ2, BZ3, BZ10	50.00%	6.67%	0.011
(vi) Tissue specimen showing microsatellite instability or deficient mismatch repair	LZ2	10.00%	3.33%	0.442

### VUSs

**Figure 2.**
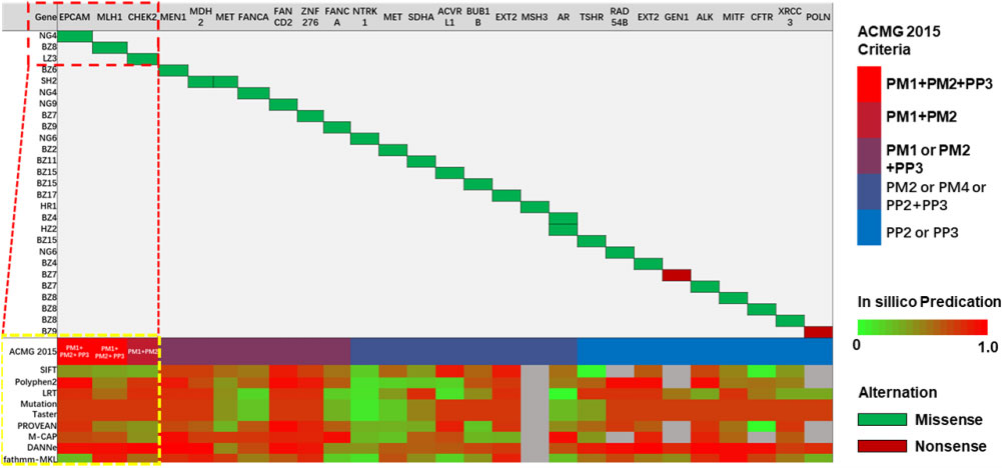
Germline profiles for 16 patients with possible high-risk VUSs. ACMG, American College of Medical Genetics. VUS, variant of unknown significance.

Among the 30 patients without any identified pathogenic/likely pathogenic mutations, 27 were found to carry a total of 129 VUSs (Supplementary Table 5). These VUSs comprised 111 missense variants, 3 frameshift variants, 3 splice-site variants, 7 non-frameshift deletions, 2 non-frameshift insertions, and 3 nonsense variants. On the basis of the American College of Medical Genetics (ACMG) 2015 guidelines [24] and *in silico* predictions by eight bioinformatic tools, 28 germline mutations in 16 patients were identified as putative high-risk VUSs (Figure 2).

### Familial pedigrees and Sanger sequencing

For the 10 probands carrying pathogenic or likely pathogenic germline variants, Sanger sequencing was performed to validate the variants in the probands as well as in their first- and second-degree relatives. The familial pedigrees of the probands with germline pathogenic or likely pathogenic mutations are shown in Figure 3.

**Figure 3.**
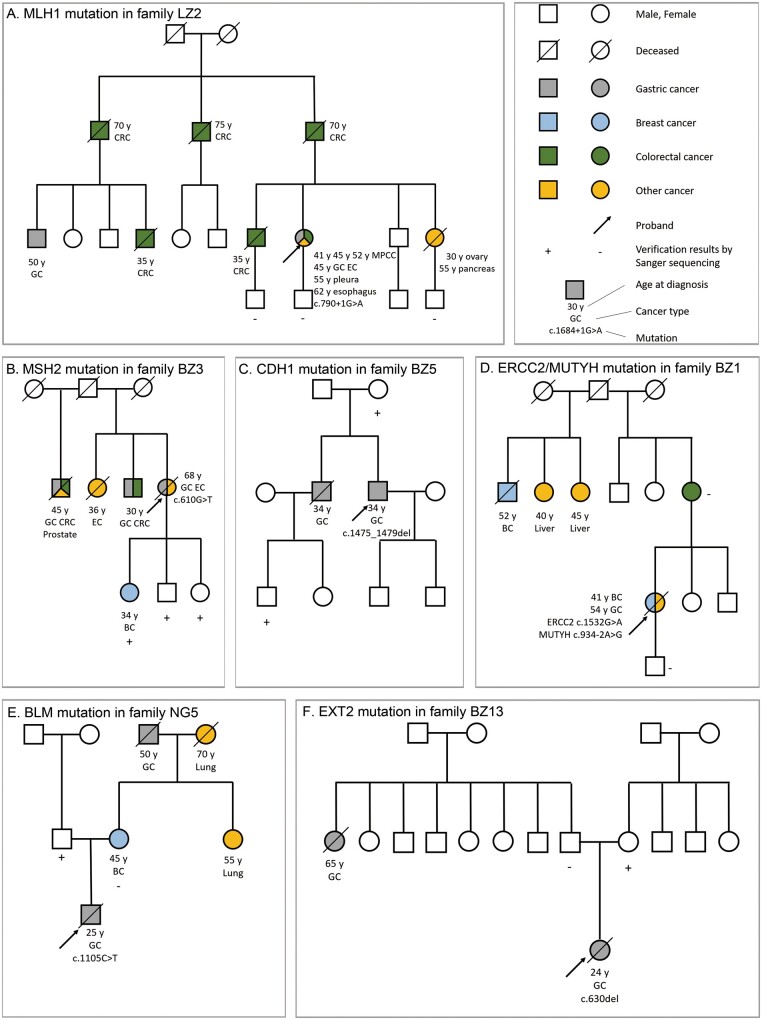
The pedigrees of probands with germline pathogenic or likely pathogenic mutations/controversial VUSs determined by next-generation sequencing. Sanger-sequencing verification is also indicated. (A) The family of LZ2 with an *MLH1* pathogenic mutation. (B) The family of BZ3 with an *MSH2* pathogenic mutation. (C) The family of BZ5 with a *CDH1* likely pathogenic mutation. (D) The family of BZ1 with an *ERCC2* likely pathogenic mutation and an *MUTYH* controversial VUS. (E) The family of NG5 with a *BLM* likely pathogenic mutation. (F) The family of BZ13 with an *EXT2* likely pathogenic mutation. (G) The family of BZ16 with a *CHEK2* likely pathogenic mutation. (H) The family of XJ1 with a *PALB2* likely pathogenic mutation. (I) The family of BZ2 with a *SPINK1* pathogenic mutation. (J) The family of BZ10 with a *SPINK1* pathogenic mutation. (K) The family of BZ14 with a *CHEK2* controversial VUS. +, mutant; −, wild-type; BC, breast cancer; CRC, colorectal cancer; EC, endometrial cancer; GC, gastric cancer; MPCC, multiple primary colorectal carcinoma; VUS, variant of unknown significance; y, age at diagnosis in years.

**Figure 3.**
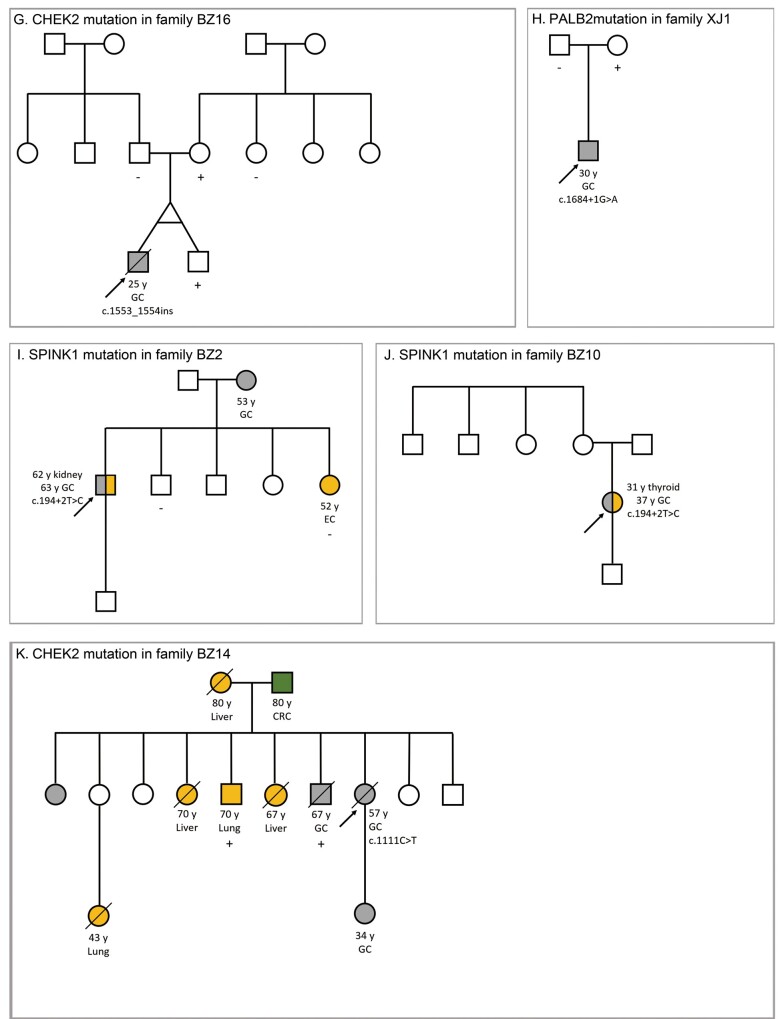
Continued.

## Discussion

Although research on hereditary GC in China is limited, investigations on other neoplasms, such as breast cancer, ovarian cancer, and prostate cancer, have suggested that the genetic spectrum of Chinese patients with hereditary tumors may differ from that of Caucasians. In general, the heterogeneous clinical features of hereditary tumor syndromes and the atypical presentations of cancer family histories hamper attempts to summarize and cluster genotypes and phenotypes using a traditional single-gene resolution approach. In contrast, this study enrolled Chinese GC patients with hereditary high risk factors for cancer, and clinical information including age at diagnosis, special histologic types, family history of malignant tumors, and microsatellite status was used to explore the frequency and spectrum of germline variants of cancer-predisposing genes. To the best of our knowledge, this is the first multicenter research study in China aiming to reveal GC-related germline variants in *CDH1* and other putative cancer-susceptibility genes through the targeted NGS of high-risk GC patients. The findings of this prospective study indicate that one in every four GC patients with hereditary high risk factors may carry pathogenic/likely pathogenic cancer-susceptibility gene variants, which were identified in nine genes: *MLH1* (*n *=* *1), *MSH2* (*n *=* *1), *CDH1* (*n *=* *1), *BLM* (*n *=* *1), *PALB2* (*n *=* *1), *EXT2* (*n *=* *1), *CHEK2* (*n *=* *1), *ERCC2* (*n *=* *1), and *SPINK1* (*n *=* *2).

The spectrum of germline variants in Chinese GC patients in this study revealed a pattern distinct from that in previous studies. Although genes such as *CDH1*, *MSH2*, and *PALB2* have been identified [25–28], there was a marked difference in the types of variants involved. Moreover, we report for the first time other mutated loci.

We identified two pathogenic variants in MMR genes associated with LS. First, the proband of the LZ2 family carried the *MLH1* splice-site variant c.790 + 1G>A, which results in the loss of amino acids 227 to 295 of the MLH1 protein and has been shown to functionally render MLH1 defective in MMR activity [29]. This variant has been reported in individuals with LS and colorectal cancer [30, 31], and multiple clinical diagnostic laboratories and reputable databases classify this variant as pathogenic. Second, the BZ3 family proband carried the *MSH2* nonsense mutation c.610G>T (p.G204*). Sheng *et al*. [32] detected this variant in one HNPCC family and classified it as a pathogenic mutation. Both of the above families met the clinical criteria for LS. The lifetime risks of LS-associated cancers, 52.0%–82.0%, are highest for colorectal cancer, followed by the risk of endometrial cancer (25.0%–60.0%), GC (6.0%–13.0), and ovarian cancer (4.0%–12.0%) [33]. Both probands developed multiple primary malignant tumors.

Clinically defined HDGC is characterized by early-onset, multigenerational diffuse GC, and lobular breast cancer. Clinical criteria for HDGC have been established by the International Gastric Cancer Linkage Consortium (IGCLC) [34]. *CDH1* is a cancer-predisposing gene that is mutated in families meeting the criteria for clinically defined HDGC, with ∼40.0% of HDGC families harboring germline mutations in *CDH1*. For example, Hansford *et al*. [27] identified 47 distinct pathogenic mutations in 183 GC patients meeting the clinical criteria for HDGC (25.7%); among these, pathogenic *CDH1* mutations were found in 31 cases. The *CDH1* germline mutation rate correlates negatively with the morbidity of GC worldwide. In countries with low GC morbidity, such as Canada, the USA, the UK, and the Netherlands, the *CDH1* mutation rate can be as high as 51.6% in patients meeting HDGC clinical criteria [35]. Conversely, the *CDH1* mutation rate is 15.4% in Japan, which has the highest GC morbidity [36]. In our study, 15 families met the HDGC clinical criteria of IGCLC2015, but only one proband carried a *CDH1* gene germline mutation (c.1475_1479delGAGTG, p. V493Sfs*42). Thus, the *CDH1* mutation rate in our study for this subset of patients was only 6.7%.

The *ERCC2* missense mutation c.1532G>A (p.R511Q) was not found in any of the databases queried. Validation of ERCC2 c.1532G>A at the functional level is, therefore, warranted. The ERCC2 p. R511Q variant is located in the region mediating interaction with MMS19, which fits the PM1 criteria. Furthermore, the frequency of this variant in GnomAD is 0, fitting the PM2 criteria; however, missense mutations of *ERCC2* result in a high rate of functional impact, which meets the PP2 criteria. On the basis of these findings, as well as the *in silico* predictions providing PP3-level evidence, we categorized this variant as likely pathogenic (PM1+PM2+PP2+PP3).

*BLM* is the causative gene of Bloom syndrome (BS). BS is an autosomal recessive disorder characterized by proportionate pre- and post-natal growth deficiency; sun sensitivity; telangiectatic, hypo-, and hyperpigmented skin; predisposition toward malignancy; and chromosomal instability [37]. This variant was classified as likely pathogenic.

The *EXT2* gene is causative of hereditary multiple exostoses (HME). HME is an autosomal dominant disorder characterized by multiple exostoses most commonly arising from the juxta-epiphyseal region of the long bones [38]. The BZ13 family patient had no symptoms associated with HME. The *EXT2* frameshift variant harbored was classified as likely pathogenic.

The *CHEK2* variant c.1553_1554insG (p.S518Rfs*7) is a well-described, lower-penetrance mutation that is mainly associated with breast cancer as well as colorectal cancer and prostate cancer. Previous studies have shown that *CHEK2* mutations predispose individuals toward GC, particularly in cases of early onset; regardless, the association between *CHEK2* and GC and the underlying molecular mechanisms require extensive investigation [39]. The frameshift variant results in the loss of almost 10% of the protein sequence, and a functional study reported that the missing region includes residues Pro515 to Pro522, which are part of a nuclear localization signal (NLS) [40]. This variant was classified as likely pathogenic.

PALB2 colocalizes with BRCA2 in the nuclear foci, promoting its localization and stability in nuclear structures and facilitating its recombinational repair and checkpoint functions. A previous study reported that *PALB2* is a breast-cancer-susceptibility gene [41]. *PALB2* mutations have also been identified in four recent studies on GC, supporting their association with GC [27, 28, 42, 43]. The *PALB2* splice-site mutation c.1684 + 1G>A results in abnormal mRNA splicing, which affects the function of the protein. This variant was detected in one patient with high-risk neuroblastoma and classified as likely pathogenic, in accordance with a previous report [44].

The *SPINK1* splice-site mutation c.194 + 2T>C was identified in the probands of the BZ2 and BZ10 families. This mutation affects a donor splice site in intron 4 of the gene: it is predicted to affect mRNA splicing, resulting in a significantly altered protein due to exon skipping, shortening, or the inclusion of intronic material. Experimental studies have shown that this splice-site variant completely abolishes *SPINK1* mRNA and protein expression in cell culture. Furthermore, this variant is recurrent in individuals of Asian descent with chronic pancreatitis [45, 46]. Multiple clinical diagnostic laboratories have, therefore, classified this variant as pathogenic. Nonetheless, the probands of the BZ2 family and BZ10 family displayed no pancreatitis symptoms.

We found a total of 129 VUSs in 27 of the patients in this study. Most of these VUSs were missense mutations; 27 VUSs were *in silico* predicted to be high-risk variants. The pathogenic classification of two of these VUSs is controversial, as described below. We employed Sanger sequencing to validate these VUSs in the two patients. Figure 3D depicts the pedigree of BZ1 with a controversial *MUTYH* VUS and Figure 3K shows the pedigree of BZ14 with a controversial *CHEK2* VUS.

MAP is an autosomal recessive disease that typically presents with multiple colorectal adenomas and an increased risk for colorectal cancer. GC among these patients is uncommon and is reported in only 2.0% of cases [47]. The proband of the BZ1 family carried the *MUTYH* splice-site mutation c.934 – 2A>G, which alters a conserved intronic nucleotide and causes aberrant splicing based on *in vitro* studies [48]. However, it remains uncertain whether this alteration causes a biological loss of function of the MUTYH protein in humans. This variant has been widely studied in East Asian populations and is frequently reported in individuals with colorectal cancer. Only one patient with GC has been described as being homozygous for this mutation; the heterozygous state was reported for all other patients harboring this variant [49–51]. Multiple clinical diagnostic laboratories and reputable databases have classified this variant as either a VUS or likely pathogenic. This conflicting evidence has prevented establishing with certainty the pathogenicity or neutrality of this variant and it was, therefore, classified in this study as a VUS.

The proband of the BZ14 family carried the *CHEK2* missense mutation c.1111C>T (p.H371Y). Liu *et al*. [52] reported that this variant confers a significantly increased risk of breast cancer in the Chinese population, though the clinical significance of this association has not yet been established. Additionally, smaller studies in Asian populations have identified this variant in breast-cancer patients as well as controls [53, 54]. According to *in vitro* functional studies, this missense mutation causes a decrease in phosphorylation and enzymatic activity compared with the wild-type CHEK2 protein. Nevertheless, the decreased activity caused by this variant is not as pronounced as the effect caused by a known kinase-disrupting variant [52]. *In silico* analyses support that this variant does not alter protein structure or function. Despite some indication that this variant may cause disease, the evidence is insufficient at present. Therefore, we classified this mutation as a VUS.

There are many differences in ethnicity, diet, and living habits between Chinese and Western populations, and clinicians in China should not simply adopt Western screening criteria for assessing hereditary GC. Thus, one of the purposes of this study was to establish screening criteria relevant to hereditary GC in China. To enroll patients, the present study referenced the clinical criteria for HDGC and LS for screening, and only patients with hereditary high risk factors were included. We found that Chinese patients with multiple-onset primary malignancies were almost 7-fold more likely to carry pathogenic germline mutations than those without such malignancies (Table 3).

Our study had several limitations. As the size of the cohort recruited was limited, we were unable to unequivocally define disease-causing variants and, therefore, GC-predisposing genes. A large number of VUSs were detected with the application of NGS, and several candidate VUSs were considered to be potentially pathogenic based on certain ACMG criteria and bioinformatic prediction tools. Further functional studies *in vitro* and *in vivo* should be performed to correctly classify these variants.

This prospective multicenter study enrolled 40 GC patients with hereditary high risk factors for cancer to explore the prevalence of germline genetic alterations in cancer-susceptibility genes by NGS. By using multiplexed panel sequencing, we found that 25.0% of patients carried pathogenic/likely pathogenic germline mutations in 9 of 171 genes tested. Several of these variants are located in GC-associated susceptibility genes or genes associated with other, clinically heterogeneous cancer-predisposition syndromes. Whether novel variants (*CHEK2*, *BLM*, *EXT2*, *ERCC2*, *PALB2*, and *SPINK1*) identified in our study confer susceptibility to GC needs to be deciphered in future work. In addition to refining the enrollment criteria to improve patient selection, future studies should focus on assessing the functional impact of all of these variants through *in vitro* testing, tumor analysis, and segregation data.

The *CDH1* gene mutation rate was 6.7% in the 15 families meeting the HDGC clinical criteria in our study, which is significantly lower than that in Western countries. This finding indicates the unique genetic background of GC in Chinese patients. Because patients with pathogenic or likely pathogenic germline variants have a dismal clinical outcome and a high rate of multicancer occurrence, it is strongly recommended to provide genetic counseling, genetic screening, and family surveillance and management for patients with hereditary high risk factors. By screening populations with hereditary high risk factors, multiple-gene sequencing can be effectively applied to discover novel disease-causing genes of hereditary disease.

## Supplementary Data

Supplementary data is available at *Gastroenterology Report* online.

## Authors’ Contributions

L.S. and X.C.W. directed, conceived of, and designed the project. Y.J.Z., Y.Y., T.X., Q.W., and S.Y.T. collected the data. Y.J.Z. drafted the manuscript. X.T.Z. and J.G. revised the manuscript. B.R.L., J.D.Z., X.B.C., Z.J.W., M.Q., X.W., and L.S. were the principal investigators from the seven institutions involved in the study. L.S. and X.C.W. supervised the study and revised the manuscript. All authors read and approved the final manuscript.

## Funding

None.

## Supplementary Material

goab020_Supplementary_Data

## References

[1] Bray F, Ferlay J, Soerjomataram I et al Global cancer statistics 2018: GLOBOCAN estimates of incidence and mortality worldwide for 36 cancers in 185 countries. CA Cancer J Clin 2018;68:394–424. 30207593 10.3322/caac.21492

[2] Zanghieri G, Di Gregorio C, Sacchetti C et al Familial occurrence of gastric cancer in the 2-year experience of a population-based registry. Cancer 1990;66:2047–51. 2224804 10.1002/1097-0142(19901101)66:9<2047::aid-cncr2820660934>3.0.co;2-g

[3] La Vecchia C, Negri E, Franceschi S et al Family history and the risk of stomach and colorectal cancer. Cancer 1992;70:50–5. 1606546 10.1002/1097-0142(19920701)70:1<50::aid-cncr2820700109>3.0.co;2-i

[4] Park YJ, Shin KH, Park JG. Risk of gastric cancer in hereditary nonpolyposis colorectal cancer in Korea. Clin Cancer Res 2000;6:2994–8. 10955776

[5] Capelle LG, Van Grieken NC, Lingsma HF et al Risk and epidemiological time trends of gastric cancer in Lynch syndrome carriers in the Netherlands. Gastroenterology 2010;138:487–92. 19900449 10.1053/j.gastro.2009.10.051

[6] Sereno M, Aguayo C, Guillén Ponce C et al Gastric tumours in hereditary cancer syndromes: clinical features, molecular biology and strategies for prevention. Clin Transl Oncol 2011;13:599–610. 21865131 10.1007/s12094-011-0705-y

[7] Oliveira C, Ferreira P, Nabais S et al E-Cadherin (CDH1) and p53 rather than SMAD4 and Caspase-10 germline mutations contribute to genetic predisposition in Portuguese gastric cancer patients. Eur J Cancer 2004;40:1897–903. 15288293 10.1016/j.ejca.2004.04.027

[8] Keller G, Vogelsang H, Becker I et al Germline mutations of the E-cadherin(CDH1) and TP53 genes, rather than of RUNX3 and HPP1, contribute to genetic predisposition in German gastric cancer patients. J Med Genet 2004;41:e89. 15173255 10.1136/jmg.2003.015594 PMC1735803

[9] van Lier MG, Westerman AM, Wagner A et al High cancer risk and increased mortality in patients with Peutz-Jeghers syndrome. Gut 2011;60:141–7. 21205875 10.1136/gut.2010.223750

[10] Giardiello FM, Brensinger JD, Tersmette AC et al Very high risk of cancer in familial Peutz-Jeghers syndrome. Gastroenterology 2000;119:1447–53. 11113065 10.1053/gast.2000.20228

[11] Giardiello FM, Trimbath JD. Peutz-Jeghers syndrome and management recommendations. Clin Gastroenterol Hepatol 2006;4:408–15. 16616343 10.1016/j.cgh.2005.11.005

[12] Jakubowska A, Nej K, Huzarski T et al BRCA2 gene mutations in families with aggregations of breast and stomach cancers. Br J Cancer 2002;87:888–91. 12373604 10.1038/sj.bjc.6600562 PMC2376177

[13] Friedenson B. BRCA1 and BRCA2 pathways and the risk of cancers other than breast or ovarian. MedGenMed 2005;7:60. PMC1681605 16369438

[14] Win AK, Hopper JL, Jenkins MA. Association between monoallelic MUTYH mutation and colorectal cancer risk: a meta-regression analysis. Fam Cancer 2011;10:1–9. 10.1007/s10689-010-9399-5 PMC3228836 21061173

[15] Iwama T, Mishima Y, Utsunomiya J. The impact of familial adenomatous polyposis on the tumorigenesis and mortality at the several organs: its rational treatment. Ann Surg 1993;217:101–8. 8382467 10.1097/00000658-199302000-00002 PMC1242747

[16] Park SY, Ryu JK, Park JH et al Prevalence of gastric and duodenal polyps and risk factors for duodenal neoplasm in Korean patients with familial adenomatous polyposis. Gut Liver 2011;5:46–51. 21461071 10.5009/gnl.2011.5.1.46 PMC3065092

[17] Lynch HT, Snyder C, Davies JM et al FAP, gastric cancer, and genetic counseling featuring children and young adults: a family study and review. Fam Cancer 2010;9:581–8. 20532639 10.1007/s10689-010-9352-7

[18] Howe JR, Mitros FA, Summers RW. The risk of gastrointestinal carcinoma in familial juvenile polyposis. Ann Surg Oncol 1998;5:751–6. 9869523 10.1007/BF02303487

[19] Pollock J, Welsh JS. Clinical cancer genetics: Part I: Gastrointestinal. Am J Clin Oncol 2011;34:332–6. 20859198 10.1097/COC.0b013e3181dea432

[20] Stanich PP, Francis DL, Sweetser S. The spectrum of findings in Cowden syndrome. Clin Gastroenterol Hepatol 2011;9:e2–3. 20655392 10.1016/j.cgh.2010.07.003

[21] Guilford P, Hopkins J, Harraway J et al E-cadherin germline mutations in familial gastric cancer. Nature 1998;392:402–5. 9537325 10.1038/32918

[22] Petrovchich I, Ford JM. Genetic predisposition to gastric cancer. Semin Oncol 2016;43:554–9. 27899187 10.1053/j.seminoncol.2016.08.006

[23] Shao D, Cheng S, Guo F et al Prevalence of hereditary breast and ovarian cancer (HBOC) predisposition gene mutations among 882 HBOC high-risk Chinese individuals. Cancer Sci 2020;111:647–57. 31742824 10.1111/cas.14242 PMC7004523

[24] Richards S, Aziz N, Bale S et al; ACMG Laboratory Quality Assurance Committee. Standards and guidelines for the interpretation of sequence variants: a joint consensus recommendation of the American College of Medical Genetics and Genomics and the Association for Molecular Pathology. Genet Med 2015;17:405–24. 25741868 10.1038/gim.2015.30 PMC4544753

[25] Slavin T, Neuhausen SL, Rybak C et al Genetic gastric cancer susceptibility in the international clinical cancer genomics community research network. Cancer Genet 2017;216–217:111–9. 10.1016/j.cancergen.2017.08.001 PMC5659836 29025585

[26] Lott PC, Carvajal-Carmona LG. Resolving gastric cancer aetiology: an update in genetic predisposition. Lancet Gastroenterol Hepatol 2018;3:874–83. 30507471 10.1016/S2468-1253(18)30237-1 PMC6500447

[27] Hansford S, Kaurah P, Li-Chang H et al Hereditary diffuse gastric cancer syndrome: CDH1 mutations and beyond. JAMA Oncol 2015;1:23–32. 26182300 10.1001/jamaoncol.2014.168

[28] Sahasrabudhe R, Lott P, Bohorquez M et al, Latin American Gastric Cancer Genetics Collaborative Group. Germline mutations in PALB2, BRCA1, and RAD51C, which regulate DNA recombination repair, in patients with gastric cancer. Gastroenterology 2017;152:983–6.e6. 28024868 10.1053/j.gastro.2016.12.010 PMC5367981

[29] Trojan J, Zeuzem S, Randolph A et al Functional analysis of hMLH1 variants and HNPCC-related mutations using a human expression system. Gastroenterology 2002;122:211–9. 11781295 10.1053/gast.2002.30296

[30] Dominguez-Valentin M, Nilbert M, Wernhoff P et al Mutation spectrum in South American Lynch syndrome families. Hered Cancer Clin Pract 2013;11:18. 24344984 10.1186/1897-4287-11-18 PMC3904200

[31] Rosty C, Clendenning M, Walsh MD, et al Colon Cancer Family Registry Cohort. Germline mutations in PMS2 and MLH1 in individuals with solitary loss of PMS2 expression in colorectal carcinomas from the Colon Cancer Family Registry Cohort. BMJ Open 2016;6:e010293. :10.1136/bmjopen-2015-010293 PMC4762074 26895986

[32] Sheng JQ, Chan TL, Chan YW et al Microsatellite instability and novel mismatch repair gene mutations in northern Chinese population with hereditary non-polyposis colorectal cancer. Chin J Dig Dis 2006;7:197–205. 17054581 10.1111/j.1443-9573.2006.00269.x

[33] Chun N, Ford JM. Genetic testing by cancer site: stomach. Cancer J 2012;18:355–63. 22846738 10.1097/PPO.0b013e31826246dc

[34] van der Post RS, Vogelaar IP, Carneiro F et al Hereditary diffuse gastric cancer: updated clinical guidelines with an emphasis on germline CDH1 mutation carriers. J Med Genet 2015;52:361–74. 25979631 10.1136/jmedgenet-2015-103094 PMC4453626

[35] Oliveira C, Senz J, Kaurah P et al Germline CDH1 deletions in hereditary diffuse gastric cancer families. Hum Mol Genet 2009;18:1545–55. 19168852 10.1093/hmg/ddp046 PMC2667284

[36] Yamada H, Shinmura K, Ito H et al Germline alterations in the CDH1 gene in familial gastric cancer in the Japanese population. Cancer Sci 2011;102:1782–8. 21777349 10.1111/j.1349-7006.2011.02038.x

[37] Kaneko H, Fukao T, Kondo N. The function of RecQ helicase gene family (especially BLM) in DNA recombination and joining. Adv Biophys 2004;38:45–64. 15493327

[38] Wuyts W, Van Hul W. Molecular basis of multiple exostoses: mutations in the EXT1 and EXT2 genes. Hum Mutat 2000;15:220–7. 10679937 10.1002/(SICI)1098-1004(200003)15:3<220::AID-HUMU2>3.0.CO;2-K

[39] Teodorczyk U, Cybulski C, Wokołorczyk D et al The risk of gastric cancer in carriers of CHEK2 mutations. Fam Cancer 2013;12:473–8. 23296741 10.1007/s10689-012-9599-2

[40] Antoni L, Sodha N, Collins I et al CHK2 kinase: cancer susceptibility and cancer therapy—two sides of the same coin? Nat Rev Cancer 2007;7:925–36. 18004398 10.1038/nrc2251

[41] Zhang F, Fan Q, Ren K et al PALB2 functionally connects the breast cancer susceptibility proteins BRCA1 and BRCA2. Mol Cancer Res 2009;7:1110–8. 19584259 10.1158/1541-7786.MCR-09-0123 PMC4928587

[42] Huang KL, Mashl RJ, Wu Y et al Pathogenic germline variants in 10,389 adult cancers. Cell 2018;173:355–70.e14. 29625052 10.1016/j.cell.2018.03.039 PMC5949147

[43] Fewings E, Larionov A, Redman J et al Germline pathogenic variants in PALB2 and other cancer-predisposing genes in families with hereditary diffuse gastric cancer without CDH1 mutation: a whole-exome sequencing study. Lancet Gastroenterol Hepatol 2018;3:489–98. 29706558 10.1016/S2468-1253(18)30079-7 PMC5992580

[44] Pugh TJ, Morozova O, Attiyeh EF et al The genetic landscape of high-risk neuroblastoma. Nat Genet 2013;45:279–84. 23334666 10.1038/ng.2529 PMC3682833

[45] Kereszturi E, Király O, Sahin-Tóth M. Minigene analysis of intronic variants in common SPINK1 haplotypes associated with chronic pancreatitis. Gut 2009;58:545–9. 18978175 10.1136/gut.2008.164947 PMC2677899

[46] Sun C, Liu M-Y, Liu X-G et al Serine Protease Inhibitor Kazal Type 1 (SPINK1) c.194 + 2T > C mutation may predict long-term outcome of endoscopic treatments in idiopathic chronic pancreatitis. Medicine (Baltimore) 2015;94:e2046. 26632706 10.1097/MD.0000000000002046 PMC5058975

[47] Vogt S, Jones N, Christian D et al Expanded extracolonic tumor spectrum in MUTYH-associated polyposis. Gastroenterology 2009;137:1976–85.e1–10. 10.1053/j.gastro.2009.08.052 19732775

[48] Tao H, Shinmura K, Hanaoka T et al A novel splice-site variant of the base excision repair gene MYH is associated with production of an aberrant mRNA transcript encoding a truncated MYH protein not localized in the nucleus. Carcinogenesis 2004;25:1859–66. 15180946 10.1093/carcin/bgh206

[49] Miyaki M, Iijima T, Yamaguchi T et al Germline mutations of the MYH gene in Japanese patients with multiple colorectal adenomas. Mutat Res 2005;578:430–3. 15890374 10.1016/j.mrfmmm.2005.01.017

[50] Kim DW, Kim IJ, Kang HC et al Germline mutations of the MYH gene in Korean patients with multiple colorectal adenomas. Int J Colorectal Dis 2007;22:1173–8. 17703316 10.1007/s00384-007-0289-8

[51] Taki K, Sato Y, Nomura S et al Mutation analysis of MUTYH in Japanese colorectal adenomatous polyposis patients. Fam Cancer 2016;15:261–5. 26684191 10.1007/s10689-015-9857-1

[52] Liu Y, Liao J, Xu Y et al A recurrent CHEK2 p.H371Y mutation is associated with breast cancer risk in Chinese women. Hum Mutat 2011;32:1000–3. 21618645 10.1002/humu.21538

[53] Chen W, Yurong S, Liansheng N. Breast cancer low-penetrance allele 1100delC in the CHEK2 gene: not present in the Chinese familial breast cancer population. Adv Ther 2008;25:496–501. 18484200 10.1007/s12325-008-0057-3

[54] Baloch AH, Daud S, Raheem N et al Missense mutations (p.H371Y, p.D438Y) in gene CHEK2 are associated with breast cancer risk in women of Balochistan origin. Mol Biol Rep 2014;41:1103–7. 24390236 10.1007/s11033-013-2956-x

