# Supplementary material for: A multicenter study assessing the prevalence of germline genetic alterations in Chinese gastric-cancer patients
Source: Gastroenterol Rep (Oxf). 2021 May 29;9(4):339–49. doi: 10.1093/gastro/goab020 (PMC8460096; doi:10.1093/gastro/goab020)
Supplement: goab020_Supplementary_Data [file goab020_supplementary_data.zip › 2020-457 Supplementary Tables 1-3.docx]

**Supplementary** **T****able 1. Geographical distribution of** **participants from 40 families**

| **Province** | **Hospital** | **Families** |
| --- | --- | --- |
| Peking | Beijing Cancer Hospital | 19 |
| Liaoning | Liaoning Cancer Hospital | 2 |
| Shanxi | Xijing Hospital | 5 |
| Nanjing | Drum Tower Hospital | 8 |
| Henan | Henan Provincial People's Hospital | 1 |
| Henan | Henan Cancer Hospital | 3 |
| Sichuan | West China Hospital | 2 |

**Supplementary Table 2. Gene selection**

| *AKT1* | *CDKN2A* | *EZH2* | *KIF1B* | *NTRK1* | *RAD51C* | *SPINK1* |
| --- | --- | --- | --- | --- | --- | --- |
| *ALK* | *CEP57* | *FAM175A* | *KIT* | *PALB2* | *RAD51D* | *SPRED1* |
| *ACVRL1* | *CFTR* | *FANCA* | *KLLN* | *PALLD* | *RAD52* | *STK11* |
| *APC* | *CHEK1* | *FANCC* | *MAX* | *PDGFRA* | *RAD54B* | *SUFU* |
| *AR* | *CHEK2* | *FANCD2* | *MC1R* | *PHB* | *RAD54 L* | *TERT* |
| *ATM* | *CTNNB1* | *FANCE* | *MDH2* | *PHOX2B* | *RB1* | *TMEM127* |
| *ATR* | *CYLD* | *FANCF* | *MEN1* | *PMS1* | *RBBP8* | *TP53* |
| *ATRX* | *DDB2* | *FANCG* | *MET* | *PMS2* | *RECQL4* | *TP53BP1* |
| *AXIN2* | *DICER1* | *FANCI* | *MITF* | *POLB* | *RET* | *TRIM24* |
| *BAP1* | *DIRC2* | *FANCL* | *MLH1* | *POLD1* | *REV3 L* | *TRIM33* |
| *BARD1* | *DIS3 L2* | *FANCM* | *MLH3* | *POLE* | *RHBDF2* | *TSC1* |
| *BLM* | *DMC1* | *FH* | *MRE11A* | *POLG* | *RNASEL* | *TSC2* |
| *BMPR1A* | *EGFR* | *FLCN* | *MSH2* | *POLH* | *RPA1* | *TSHR* |
| *BRCA1* | *ELAC2* | *GALNT12* | *MSH3* | *POLN* | *RPS20* | *VHL* |
| *BRCA2* | *EMSY* | *GEN1* | *MSH6* | *POLQ* | *SDHA* | *WRN* |
| *BRIP1* | *EME1* | *SLX1A* | *MSR1* | *POT1* | *SDHAF2* | *WT1* |
| *BUB1* | *EME2* | *SLX1B* | *MUS81* | *PPP2R2A* | *SDHB* | *XPA* |
| *BUB1B* | *ENG* | *GPC3* | *MUTYH* | *PPM1D* | *SDHC* | *XPC* |
| *CDC73* | *EPCAM* | *GREM1* | *MXI1* | *PRKDC* | *SDHD* | *XRCC2* |
| *CDH1* | *ERCC2* | *HMMR* | *NBN* | *PRSS1* | *SHFM1* | *XRCC3* |
| *CDK4* | *ERCC3* | *HNF1A* | *NCOA4* | *PTCH1* | *SLX4* | *RAD51* |
| *CDK6* | *ERCC4* | *HNF1B* | *NF1* | *PTCH2* | *SMAD4* |  |
| *CDK12* | *ERCC5* | *EPAS1* | *NF2* | *PTEN* | *SMARCA4* |  |
| *CDKN1B* | *EXT1* | *HOXB13* | *NQO2* | *RAD50* | *SMARCB1* |  |
| *CDKN1C* | *EXT2* | *HRAS* | *NTHL1* | *RAD51B* | *SMARCE1* |  |

**Supplementary Table 3.** **Detailed information for patients meeting the enrollment criteria**

| Family code | Cancer | Age at GC diagnosis | Stage | Family history of cancer | Criteria* | | | | | |
| --- | --- | --- | --- | --- | --- | --- | --- | --- | --- | --- |
|  |  |  |  |  | 1 | 2 | 3 | 4 | 5 | 6 |
| SH1 | GC | 54 | I | Yes |  |  |  | **√** |  |  |
| SH2 | GC | 30 | I | Yes | **√** |  |  |  |  |  |
| NG1 | GC | 29 | III | No | **√** |  |  |  |  |  |
| NG2 | GC | 27 | III | Yes | **√** |  |  |  |  |  |
| NG3 | GC | 29 | III | Yes | **√** |  | **√** | **√** |  |  |
| NG4 | GC | 28 | II | Yes | **√** |  |  |  |  |  |
| **NG5** | **GC** | **25** | IV | **Yes** | **√** |  | **√** | **√** |  |  |
| NG6 | GC | 55 | III | Yes |  |  |  | **√** |  |  |
| NG7 | GC | 38 | IV | Yes |  |  | **√** | **√** |  |  |
| NG8 | GC | 26 | III | No | **√** |  |  |  |  |  |
| NG9 | GC | 34 | III | No |  | **√** |  |  |  |  |
| HR1 | GC | 36 | III | Yes |  |  | **√** |  |  |  |
| HZ1 | GC | 51 | I | Yes |  |  |  | **√** |  |  |
| HZ2 | GC | 40 | IV | Yes |  |  | **√** |  |  |  |
| **XJ1** | **GC** | **30** | II | **No** | **√** |  |  |  |  |  |
| XJ2 | GC | 29 | IV | No | **√** |  |  |  |  |  |
| XJ3 | GC | 29 | II | No | **√** |  |  |  |  |  |
| XJ4 | GC | 32 | III | No |  | **√** |  |  |  |  |
| LZ1 | GC | 57 | III | Yes |  |  |  | **√** |  |  |
| **LZ2** | **MPCC, EC, pleural fibrosarcoma, esophageal leiomyosarcoma** | **45** | III | **Yes** |  |  | **√** | **√** | **√** | **√** |
| LZ3 | GC, CRC | 63 | III | Yes |  |  |  | **√** | **√** |  |
| **BZ1** | **GC, BC** | **54** | III | **Yes** |  |  |  | **√** | **√** |  |
| **BZ2** | **GC, renal cancer** | **63** | III | **Yes** |  |  |  | **√** | **√** |  |
| **BZ3** | **GC, EC** | **68** | IV | **Yes** |  |  |  | **√** | **√** |  |
| BZ4 | GC | 30 | I | No | **√** |  |  |  |  |  |
| **BZ5** | **GC** | **34** | II | **Yes** |  |  | **√** |  |  |  |
| BZ6 | GC | 53 | IV | Yes |  |  |  | **√** |  |  |
|  |  |  | II |  |  |  |  |  |  |  |
| BZ7 | GC | 27 | II | No | **√** |  |  |  |  |  |
| BZ8 | GC | 28 | II | Yes | **√** |  |  |  |  |  |
| BZ9 | GC, lung adenocarcinoma, GC | 60 | III | Yes |  |  |  | **√** | **√** |  |
| **BZ10** | **GC, thyroid cancer** | **37** | IV | **No** |  |  |  |  | **√** |  |
| BZ11 | GC | 53 | III | Yes |  |  |  | **√** |  |  |
| BZ12 | GC | 59 | IV | Yes |  |  |  | **√** |  |  |
| **BZ13** | **GC** | **24** | I | **Yes** | **√** |  |  |  |  |  |
| BZ14 | GC | 60 | IV | Yes |  |  |  | **√** |  |  |
| BZ15 | GC | 48 | IV | Yes |  |  | **√** | **√** |  |  |
| **BZ16** | **GC** | **26** | III | **No** | **√** |  |  |  |  |  |
| BZ17 | GC | 76 | I | Yes |  |  |  | **√** |  |  |
| BZ18 | GC | 61 | II | Yes |  |  |  | **√** |  | **√** |
| BZ19 | GC | 59 | I | Yes |  |  |  | **√** |  |  |
| **Total** |  |  |  |  | **4/15** | **0/2** | **3/8** | **5/21** | **5/7** | **1/2** |

*Criteria for high risk were included: 1) onset age ≤ 30 years, regardless of family history; 2) onset age ≤ 35 years and GC histologically classified as signet ring cell carcinoma (SRCC) or mucinous adenocarcinoma, regardless of family history; 3) onset age ≤ 50 years and at least one first-degree relative diagnosed with malignant tumors; 4) at least two first- or second-degree relatives diagnosed with malignant tumors, with at least one first-degree relative included; 5) diagnosed with more than two primary malignant tumors, with one having an onset age ≤ 50 years; 6) tissue specimens showing microsatellite instability or deficient mismatch repair.

GC: gastric cancer; MPCC: multiple primary colorectal carcinoma, CRC: colorectal cancer; EC: endometrial cancer; BC: breast cancer

**Families** **shown in bold** are patients with pathogenic/likely pathogenic variants. The red or pink boxes highlight which enrollment criteria these 40 patients met in this study.

**Supplementary Table 4.**

| No. | Family code | Sex | Age at GC diagnosis | Differentiation degree | Histologic types | Lauren classification | HER2 status | TNM | Stage | History of other primary tumors | Family history of cancer | history of cancer in first degree relatives | history of cancer in second degree relatives | Time to First diagnosis | Time to death / final follow-up |
| --- | --- | --- | --- | --- | --- | --- | --- | --- | --- | --- | --- | --- | --- | --- | --- |
| 1 | SH1 | Male | 54 | Highly | tubular adenocarcinoma | intestinal type | unknown | T1N0M0 | I | No | Yes | Yes | Yes | 2016/11/22 | 2019/1/11 |
| 2 | SH2 | Female | 30 | poorly | signet-ring cell carcinoma | diffuse type | unknown | T2N0M0 | I | No | Yes | Yes | Yes | 2017/1/3 | 2019/1/11 |
| 3 | NG1 | Female | 29 | poorly | signet-ring cell carcinoma | diffuse type | 1+ | T3N3M0 | III | No | No | No | No | 2016/5/6 | 2017/9/12 |
| 4 | NG2 | Male | 27 | poorly | signet-ring cell carcinoma | diffuse type | 1+ | T4aN3M0 | III | No | Yes | Yes | No | 2017/1/11 | 2017/12/4 |
| 5 | NG3 | Male | 29 | poorly | signet-ring cell carcinoma | diffuse type | 2+ | T3N2M0 | III | No | Yes | Yes | Yes | 2017/1/4 | 2018/11/6 |
| 6 | NG4 | Male | 28 | poorly | adenocarcinoma | unknown | unknown | T1bN1M0 | IIA | No | Yes | No | Yes | 2017/1/26 | 2018/11/6 |
| 7 | NG5 | Male | 25 | poorly | adenocarcinoma | mixed type | unknown | T4bN3aM1 | IV | No | Yes | Yes | Yes | 2017/1/8 | 2018/11/6 |
| 8 | NG6 | Male | 55 | poorly | adenocarcinoma | unknown | 0 | T3N3bM0 | IIIC | No | Yes | Yes | Yes | 2017/1/27 | 2018/6/22 |
| 9 | NG7 | Female | 38 | poorly | signet-ring cell carcinoma | unknown | unknown | T4aN0M1 | IV | No | Yes | No | Yes | 2017/2/20 | 2018/10/1 |
| 10 | NG8 | Female | 26 | poorly | signet-ring cell carcinoma | diffuse type | 0 | T4aN1M0 | IIIA | No | No | No | No | 2017/2/23 | 2018/11/6 |
| 11 | NG9 | Female | 34 | poorly | signet-ring cell carcinoma | unknown | unknown | T4aN2M1 | IIIA | No | No | No | No | 2016/6/16 | 2017/11/15 |
| 12 | HR1 | Male | 36 | poorly | adenocarcinoma | unknown | unknown | T4N2M0 | IIIA | No | Yes | Yes | No | 2017/3/30 | 2019/1/11 |
| 13 | HZ1 | Male | 51 | poorly | adenocarcinoma | unknown | unknown | T1N0M0 | I | No | Yes | Yes | No | 2016/9/14 | 2019/1/11 |
| 14 | HZ2 | Male | 40 | poorly | adenocarcinoma | unknown | unknown | T3N1M1 | IV | No | Yes | Yes | No | 2016/12/15 | 2017/9/13 |
| 15 | LZ1 | Male | 57 | poorly | adenocarcinoma | unknown | unknown | T4aN2M0 | IIIB | No | Yes | Yes | No | 2016/10/27 | 2019/1/11 |
| 16 | LZ2 | Female | 45 | Highly | adenocarcinoma | unknown | unknown | T4N2M0 | III | Yes | Yes | Yes | No | 2001/4/2 | 2019/1/11 |
| 17 | LZ3 | Male | 63 | moderately | adenocarcinoma | unknown | unknown | T4N2M0 | III | Yes | Yes | Yes | No | 2015/12/1 | 2019/1/11 |
| 18 | XJ1 | Male | 30 | poorly | adenocarcinoma | diffuse type | 0 | T3N0M0 | IIB | No | No | No | No | 2017/4/18 | 2018/10/15 |
| 19 | XJ2 | Female | 29 | poorly | adenocarcinoma | unknown | 0 | T4bN+M1 | IV | No | No | No | No | 2017/5/18 | 2018/2/9 |
| 20 | XJ3 | Female | 29 | poorly | adenocarcinoma | diffuse type | 3+ | T3N1M0 | IIB | No | No | No | No | 2017/7/25 | 2018/11/15 |
| 21 | XJ4 | Male | 30 | poorly | adenocarcinoma | diffuse type | 0 | T4aN3aM0 | IIIB | No | No | No | No | 2017/6/20 | 2018/10/24 |
| 22 | BZ1 | Female | 54 | poorly | adenocarcinoma | unknown | 2+，no amplification in FISH | T4N3bM0 | IIIB | Yes | Yes | Yes | Yes | 2014/8/15 | 2016/12/17 |
| 23 | BZ2 | Male | 63 | poorly | mucinous adenocarcinoma | unknown | unknown | T4aN3M0 | III | Yes | Yes | Yes | No | 2016/9/13 | 2018/12/19 |
| 24 | BZ3 | Female | 68 | poorly | signet-ring cell carcinoma | unknown | unknown | T4bN+M1 | IV | Yes | Yes | Yes | Yes | 2016/8/15 | 2019/1/11 |
| 25 | BZ4 | Female | 30 | poorly | adenocarcinoma with signet ring cells | unknown | 0 | pT1N0M0 | I | No | No | No | No | 2015/3/13 | 2018/11/22 |
| 26 | BZ5 | Male | 34 | poorly | adenocarcinoma | diffuse type | 0 | pT3N1M0 | IIB | No | Yes | Yes | No | 2012/2/1 | 2019/1/11 |
| 27 | BZ6 | Female | 53 | poorly | adenocarcinoma | diffuse type | 2+，no amplification in FISH | T4N2M1 | IV | No | Yes | Yes | No | 2017/7/21 | 2019/1/2 |
| 28 | BZ7 | Male | 27 | poorly | adenocarcinoma with signet ring cells | diffuse type | 0 | T4aN0M0 | IIB | No | No | No | No | 2012/5/23 | 2018/2/28 |
| 29 | BZ8 | Male | 28 | poorly | adenocarcinoma | diffuse type | 0 | T4N0M0 | IIB | No | Yes | No | Yes | 2017/5/1 | 2019/1/11 |
| 30 | BZ9 | Male | 60 | poorly | adenocarcinoma | unknown | 1+ | T2N1M0 | II | Yes | Yes | Yes | Yes | 2011/1/1 | 2019/1/11 |
| 31 | BZ10 | Female | 37 | poorly | adenocarcinoma with signet ring cells | diffuse type | 1+ | T4aN1M0 | IIIA | Yes | No | No | No | 2015/7/1 | 2019/1/2 |
| 32 | BZ11 | Male | 53 | poorly | adenocarcinoma with signet ring cells | diffuse type | 1+ | T2N0M1 | IV | No | Yes | Yes | No | 2016/8/26 | 2018/11/7 |
| 33 | BZ12 | Female | 59 | poorly | signet-ring cell carcinoma | unknown | 0 | T3N2M0 | IIIA | No | Yes | Yes | Yes | 2017/9/13 | 2019/1/11 |
| 34 | BZ13 | Female | 24 | poorly | adenocarcinoma | mixed type | 1+ | TxN3M1 | IV | No | Yes | No | Yes | 2017/5/1 | 2017/9/15 |
| 35 | BZ14 | Female | 60 | poorly | adenocarcinoma | unknown | unknown | T2bN0M0 | IB | No | Yes | Yes | No | 2014/6/15 | 2019/1/11 |
| 36 | BZ15 | Male | 48 | poorly | signet-ring cell carcinoma | diffuse type | unknown | T4bN2M1 | IV | No | Yes | Yes | Yes | 2017/12/6 | 2019/1/11 |
| 37 | BZ16 | Male | 26 | poorly | adenocarcinoma | unknown | 0 | T4bNxM1 | IV | No | No | No | No | 2017/12/15 | 2018/6/1 |
| 38 | BZ17 | Male | 76 | poorly | adenocarcinoma with signet ring cells | mixed type | 0 | T4aN1M0 | IIIA | No | Yes | Yes | No | 2018/1/30 | 2019/1/11 |
| 39 | BZ18 | Female | 60 | poorly | tubular adenocarcinoma | intestinal type | 0 | T2N0M0 | IB | No | Yes | Yes | Yes | 2018/2/13 | 2019/1/11 |
| 40 | BZ19 | Male | 59 | poorly | adenocarcinoma | unknown | 0 | T4N0M0 | IIB | No | Yes | Yes | Yes | 2018/8/20 | 2019/1/11 |
